# MIND diet score and its association with metabolic dysfunction-associated steatotic liver disease and gut microbiota profiles: a cross-sectional study

**DOI:** 10.3389/fnut.2025.1637572

**Published:** 2025-08-25

**Authors:** Shengbo Yang, Litao Zhang, Mingli Hu, Weina He

**Affiliations:** ^1^Hepatobiliary Pancreatic Spleen Surgery, The Second Affiliated Hospital of Guizhou University of Traditional Chinese Medicine, Guiyang City, Guizhou Province, China; ^2^Emergency Department, The Second Affiliated Hospital of Guizhou University of Traditional Chinese Medicine, Guiyang City, Guizhou Province, China

**Keywords:** MIND diet, metabolic dysfunction-associated steatotic liver disease (MASLD), gut microbiota, insulin resistance, diet, fibrosis, hepatic steatosis, inflammation

## Abstract

**Background:**

Metabolic dysfunction-associated steatotic liver disease (MASLD) is a rising health issue linked to poor diet and gut microbiota dysbiosis. The Mediterranean-DASH Intervention for Neurodegenerative Delay (MIND) diet, high in polyphenols and anti-inflammatory nutrients, may help protect against MASLD. This study examined how adherence to the MIND diet relates to MASLD severity, focusing on hepatic steatosis, fibrosis, insulin resistance, inflammation, and gut microbiota diversity.

**Methods:**

A cross-sectional analysis of 450 adults with confirmed MASLD was conducted. MIND diet scores were derived from a validated FFQ. Hepatic outcomes were assessed via ultrasonography, hepatic steatosis index (HSI), controlled attenuation parameter (CAP), and fibrosis-4 (FIB-4) index. Biomarkers included liver enzymes (ALT, AST, GGT, and ALP), HOMA-IR, lipid profile, C-reactive protein (CRP), lipopolysaccharide (LPS), and trimethylamine N-oxide (TMAO). Gut microbiota composition was analyzed using 16S rRNA sequencing.

**Results:**

Higher MIND scores were associated with reduced steatosis as measured by HSI and CAP, lower fibrosis indicated by the FIB-4 index, improved insulin sensitivity reflected by lower HOMA-IR values, decreased liver enzyme levels, and a more favorable lipid profile. Inflammatory markers (CRP, LPS) decreased with higher adherence (all *p* < 0.001). Each 1-unit increase in MIND diet score was significantly associated with reductions in fasting insulin (β = −0.20 μU/mL; *p* < 0.001), HOMA-IR (β = −2.11; *p* < 0.001), CRP (β = −2.12; *p* < 0.001), LPS (β = −8.52; *p* < 0.001). Notably, Simpson diversity index scores were higher, and the Firmicutes-to-Bacteroidetes ratio (F/B ratio) was lower, among participants with greater MIND adherence, reflecting improved microbial balance. Mediation analysis revealed that Simpson diversity partially mediated the relationship between MIND score and liver health indicators, suggesting a role for gut microbial diversity in modulating hepatic outcomes.

**Conclusion:**

Greater adherence to the MIND diet was associated with indicators of better liver function, lower systemic inflammation, and improved metabolic health, and a more favorable gut microbiota composition in adults with MASLD.

## Introduction

Metabolic dysfunction-associated steatotic liver disease (MASLD), formerly termed non-alcoholic fatty liver disease (NAFLD), is the most common chronic liver disorder globally, closely linked to the increasing prevalence of obesity, insulin resistance, dyslipidemia, and metabolic syndrome (1, 2). MASLD encompasses a continuum of liver pathology ranging from simple steatosis to metabolic dysfunction-associated steatohepatitis (MASH), progressive fibrosis, cirrhosis, and hepatocellular carcinoma (3). Despite its substantial health burden, no pharmacological therapies have yet been formally approved for MASLD, reinforcing the urgent need for effective dietary and lifestyle interventions that address its underlying metabolic and inflammatory drivers (4).

Dietary patterns play a pivotal role in the progression and development of MASLD (5, 6). The Mediterranean-DASH Intervention for Neurodegenerative Delay (MIND) diet, a combination of the Mediterranean and DASH (Dietary Approaches to Stop Hypertension) eating plans, has shown protective effects on the brain, cardiovascular system, and metabolism, along with anti-inflammatory properties (7, 8). The MIND diet emphasizes high consumption of green leafy vegetables, berries, nuts, legumes, whole grains, fish, and olive oil, while limiting red meat, butter, cheese, pastries, and fast food (9). These components are rich in polyphenols, antioxidants, and anti-inflammatory nutrients that may modulate hepatic lipid metabolism, insulin signaling, and oxidative stress—hallmarks of MASLD pathogenesis (10).

Emerging evidence highlights the critical role of the gut-liver axis in MASLD development. Alterations in gut microbiota composition, referred to as dysbiosis, have been associated with enhanced intestinal permeability, translocation of bacterial endotoxins such as lipopolysaccharide (LPS), induction of systemic inflammation, and hepatic lipid accumulation (11). Diet is a major determinant of gut microbial structure and function, and it is increasingly recognized that the beneficial effects of certain dietary patterns on liver health may be mediated, at least in part, through modulation of gut microbiota (12).

However, there is a critical gap in the literature regarding the specific impact of the MIND diet on MASLD severity and the mediating role of gut microbiota profiles in this relationship. While several studies have explored links between the Mediterranean or DASH diets and liver-related outcomes (13, 14), research examining the MIND diet's role in hepatic steatosis and fibrosis—especially through microbiota-mediated pathways—is scarce. Furthermore, prior investigations often lack comprehensive microbiome assessments using advanced sequencing technologies or fail to evaluate potential microbial mediators linking diet and disease severity.

This study addresses these gaps by comprehensively evaluating the association between adherence to the MIND diet and MASLD severity, incorporating clinically relevant hepatic outcomes, systemic inflammation, and detailed gut microbiota profiles. We hypothesize that higher adherence to the MIND diet will be associated with reduced hepatic steatosis and fibrosis, improved metabolic profiles, lower systemic inflammation, and enhanced gut microbial diversity. The primary aim of this study is to investigate whether gut microbiota diversity and composition mediate the relationship between MIND diet adherence and the clinical severity of MASLD. By employing mediation analysis, we further explore the extent to which gut microbial α-diversity serves as a mechanistic pathway linking dietary quality to hepatic and metabolic health.

## Materials and methods

### Study design and participants

**Figure 1:**
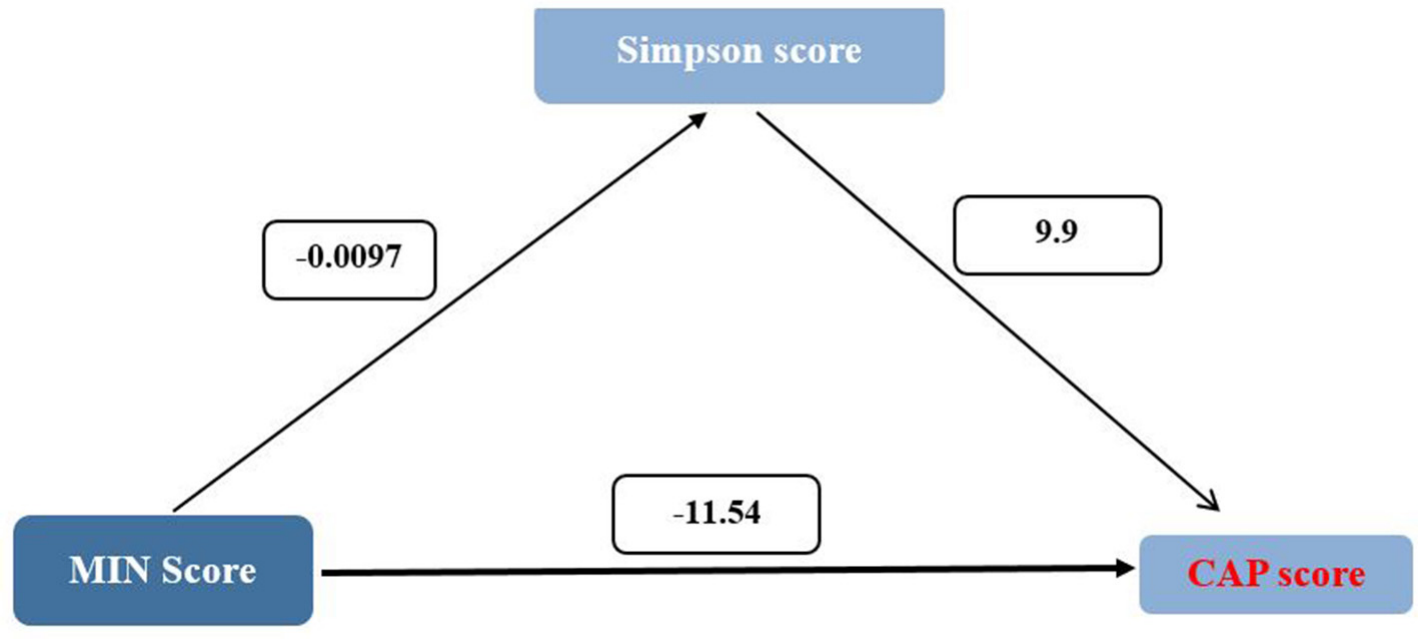
Flowchart of study participants' enrollment and inclusion. Flowchart illustrating the selection and inclusion process of the 450 participants from the initial 520 screened individuals, detailing reasons for exclusion.

This study employed a cross-sectional design to investigate the association between adherence to the MIND diet and the severity of MASLD, including hepatic, inflammatory, cardiometabolic, and gut microbiota profiles. This cross-sectional study was conducted between January 2022 and December 2023 at the outpatient hepatology clinic of The Second Affiliated Hospital of Guizhou University of Traditional Chinese Medicine, a tertiary care center specializing in liver disease management in Guiyang City, Guizhou Province, China. Participants were adults aged 18 to 70 years, selected to encompass a broad adult population diagnosed with MASLD. To minimize recall bias—particularly among older individuals—dietary data were collected through interviewer-administered questionnaires, supported by visual aids, and interviews were conducted shortly after food consumption whenever possible. MASLD was defined based on evidence of hepatic steatosis identified via transabdominal ultrasonography or non-invasive scoring systems (e.g., Hepatic Steatosis Index [HSI]), in the absence of significant alcohol consumption (>140 g/week for men and >70 g/week for women) or alternative causes of steatosis. Exclusion criteria included a diagnosis of cirrhosis (Child-Pugh B or C), viral hepatitis, autoimmune liver disease, cancer, pregnancy, bariatric surgery, and severe comorbidities (e.g., heart failure or renal impairment). Additionally, participants were excluded if they had used antibiotics, probiotics, or corticosteroids within the three months prior to enrollment. Primary outcomes included hepatic steatosis severity, fibrosis stage (FIB-4 index), liver enzyme levels (ALT, AST, GGT), markers of systemic inflammation (CRP, LPS, TMAO), and metabolic parameters (HOMA-IR, lipid profile). Secondary outcomes included F/B ratio and Simpson index. Efforts were made to minimize bias through standardized protocols for data collection and blinded assessment of ultrasound results. Recall bias in dietary reporting was mitigated by using a validated FFQ and trained interviewers. Selection bias may be present due to recruitment from a single center, which is acknowledged in the limitations section. Of the 520 individuals initially screened, 70 were excluded due to missing data, cirrhosis, or exclusion criteria. Final analysis included 450 participants (Flowchart, Figure 1).

### Sample size

Sample size was determined based on power analysis from previous studies (15), assuming a moderate effect size (β ≥ 0.2) with 90% power and a significance level of α = 0.05, accounting for multiple biomarker comparisons, resulting in a final sample size of 450.

### Ethics approval

The study protocol was approved by the Institutional Review Board of Medical Ethics Committee of the Second Affiliated Hospital of Guizhou University of Traditional Chinese Medicine under reference number No.20221005-42. All participants provided written informed consent prior to enrollment. All procedures were conducted in accordance with the ethical principles outlined in the Declaration of Helsinki. Data were anonymized and handled confidentially in compliance with institutional policies on data protection and participant privacy.

### Dietary assessment and MIND scoring

Dietary intake was assessed using a validated semi-quantitative food frequency questionnaire (FFQ), administered by trained dietitians, which has been widely used and validated in similar Chinese populations (16). Participants reported habitual intake over the past year, with portion sizes and consumption frequencies standardized. This included 10 brain-healthy food groups (green leafy vegetables, other vegetables, nuts, berries, legumes, whole grains, fish, poultry, olive oil, and wine) and 5 brain-unhealthy groups (red meats, butter/margarine, cheese, pastries/sweets, and fast/fried food). Scoring followed established protocols, yielding a total score ranging from 0 to 15, where higher scores indicate greater adherence (17). Dietary intakes were energy-adjusted via the residual method. Participants were categorized into tertiles per component. For brain-healthy foods, the highest tertile scored 1, middle 0.5, and lowest 0. For brain-unhealthy foods, scoring was reversed (highest tertile = 0, lowest = 1). Component scores were summed to yield a total MIND score ranging from 0 (lowest adherence) to 15 (highest). For analysis, scores < 8.5 indicated low adherence, and ≥9 indicated high adherence (18). Detailed scoring methodology, frequency options, and example calculations for both low- and high-adherence participants are provided in Supplementary material 1.

### Hepatic, metabolic, and inflammatory assessments

Hepatic steatosis was evaluated using transabdominal ultrasonography performed by experienced radiologists blinded to clinical data. Steatosis severity was graded as mild, moderate, or severe based on standard sonographic features including liver echogenicity, visualization of intrahepatic vessels, and diaphragm delineation. Additionally, the Hepatic Steatosis Index (HSI) and Controlled Attenuation Parameter (CAP) were used as continuous measures of hepatic fat accumulation. Fibrosis was estimated using the Fibrosis-4 (FIB-4) index, which incorporates age, platelet count, AST, and ALT levels. FIB-4 values below 1.3 were considered to rule out advanced fibrosis, whereas values above 2.67 indicated a high likelihood of significant fibrosis (19).

Venous blood samples were obtained following an overnight fast. Serum levels of alanine aminotransferase (ALT), aspartate aminotransferase (AST), gamma-glutamyl transferase (GGT), and alkaline phosphatase (ALP) were assessed as biochemical markers of hepatic injury. Metabolic parameters evaluated included fasting glucose, fasting insulin, and lipid profile components—namely total cholesterol (TC), triglycerides (TG), low-density lipoprotein cholesterol (LDL-C), and high-density lipoprotein cholesterol (HDL-C). Insulin resistance was estimated using the homeostasis model assessment (HOMA-IR). Systemic inflammation was evaluated via high-sensitivity C-reactive protein (CRP) levels, whereas serum concentrations of lipopolysaccharide (LPS) and trimethylamine N-oxide (TMAO) were measured as indicators of metabolic endotoxemia and gut-derived metabolite production, respectively.

Physical activity levels were monitored using the International Physical Activity Questionnaire (IPAQ), while body weight was measured without shoes using a scale accurate to 0.250 kg (Seca, Hamburg, Germany), height was assessed without shoes with a measuring tape accurate to 0.5 cm, and BMI was calculated by dividing weight (kg) by height squared (m^2^). Systolic blood pressure (SBP) and diastolic blood pressure (DBP) were measured using an automatic oscillometric device (Omron Healthcare Co., Ltd) following a 5-min rest period.

### Gut microbiota profiling

Fecal samples were self-collected by participants using standardized sterile collection kits, immediately stored at −80°C to preserve microbial DNA integrity, and processed at The Second Affiliated Hospital of Guizhou University of Traditional Chinese Medicine. Microbial DNA was extracted using the QIAamp PowerFecal Pro DNA Kit (Qiagen, Catalog No. 51884) following manufacturer protocols, including bead-beating for effective cell lysis. The V3–V4 hypervariable region of the bacterial 16S rRNA gene was PCR-amplified and sequenced on the Illumina MiSeq platform with paired-end reads. Raw sequencing data underwent quality control using the QIIME2 pipeline, including trimming of low-quality bases, removal of chimeric sequences, and denoising with DADA2 to generate high-quality amplicon sequence variants (ASVs). Data analysis was performed using QIIME2 (version 2022.8) for quality control and taxonomic classification, with DADA2 (version 1.22) for denoising and generation of amplicon sequence variants (ASVs). Taxonomic classification was assigned using the SILVA reference. Taxonomic classification was assigned using the SILVA reference. To comprehensively evaluate the microbial community diversity within each sample, alpha-diversity indices were calculated, including the Shannon and Simpson metrics (20). The Shannon index accounts for both species richness (the total number of unique taxa) and their relative abundance, providing a measure sensitive to rare species, while the Simpson index emphasizes the evenness or dominance of species, reflecting the probability that two randomly selected individuals belong to different taxa. Together, these indices offer complementary insights into the complexity and balance of the gut microbiota. Additionally, the Firmicutes-to-Bacteroidetes (F/B) ratio—a widely recognized biomarker of gut microbial composition and dysbiosis—was computed by comparing the relative abundance of these two predominant bacterial phyla, which have been implicated in metabolic and inflammatory processes (21).

### Statistical analysis

Descriptive statistics characterized the population by MIND diet score quartiles, with normally distributed variables summarized as mean ± SD, skewed variables as median (IQR), and categorical variables as frequencies (%). Linear regression models evaluated the association between standardized MIND scores and outcomes of interest, with Model 1 adjusting for age, sex, BMI, and physical activity (MET-min/week), and Model 2 further adjusting for total calorie and fiber intake. To assess clinical relevance, regression coefficients (β) were evaluated against a threshold of |β| ≥ 0.2, based on existing literature and expert interpretation. Given the multiple comparisons in regression analyses, a Bonferroni correction was applied, adjusting *p*-values by multiplying by the number of comparisons (*n* = 20), resulting in a significance threshold of *p* < 0.0025, with adjusted *p*-values above 1 capped at 1.0 to ensure conservative interpretation. A generalized structural equation model (GSEM) was employed to estimate direct and indirect effects while accounting for potential mediation, with all variables standardized for comparability and maximum likelihood estimation used to derive path coefficients. Additionally, multivariate regression analysis evaluated the relationship between standardized independent variables, including FIB4, CAP, HSI, and Hepatic Steatosis, and the MIND score, utilizing z-scores for adjusted standardization to control for confounding factors.

## Results

### Participant characteristics by MIND diet score quartiles

Table 1 presents the baseline sociodemographic, anthropometric, and dietary characteristics of the 450 participants stratified by quartiles of MIND diet score (Q1: lowest adherence; Q4: highest adherence). The mean age of the cohort was 59.29 ± 10.81 years, with no statistically significant differences across quartiles (*p* = 0.117). There was also no significant difference in sex distribution (*p* = 0.483), with females comprising 46.5% of the total sample. Mean MIND scores increased significantly from Q1 to Q4 (*p* < 0.001), ranging from 3.70 ± 1.10 in the lowest quartile to 12.89 ± 0.89 in the highest. No significant differences were observed in socioeconomic status or physical activity levels (MET-minutes/week) across groups (*p* > 0.05). Participants in higher MIND score quartiles had lower body BMI and waist circumference (WC), with statistically significant differences observed for both variables (BMI: *p* < 0.001; WC: *p* < 0.001). Total energy intake did not differ significantly across quartiles (*p* = 0.511); however, fiber intake increased significantly with higher MIND adherence (*p* < 0.001). Consumption of brain-healthy foods—including green leafy vegetables, other vegetables, nuts, olive oil, and whole grains—was significantly higher among participants in higher MIND score quartiles (all *p* < 0.001), reflecting greater adherence to the diet pattern.

**Table 1:** Characteristics of study participants by MIND score quartiles (*N* = 450).

**Variables**	**Total (*N* = 450)**	**Q1 (*N* = ‘131)**	**Q2 (*N* = 112)**	**Q3 (*N* = 96)**	**Q4 (*N* = 111)**	***P*-value**
MIND (mean) mean ± SD	8.22 ± 3.70	3.70 ± 1.10	7.01 ± 0.90	10.28 ± 0.83	12.98 ± 0.89	< 0.001
Age (years) mean ± SD	59.29 ± 10.81	60.02 ± 8.87	59.42 ± 4.79	57.00 ± 9.49	60.29 ± 16.63	0.117
Sex, Female, n (%)	209 (46.5)	65 (31)	45 (21)	46 (22)	53 (24.5)	0.483
**Socio-Economic Status (SES)**						
Low	49 (36)	49 (37)	46 (41.5)	32 (33)	35 (32)	0.724
Medium	28 (23)	28 (21)	24 (21.5)	21 (22)	30 (27)	
High	54 (41)	54 (41)	41 (37)	43 (45)	45 (41)	
Physical activity (total MET)	562.97 ± 47.49	562.13 ± 47.55	566.03 ± 46.58	562.65 ± 48.99	561.13 ± 47.51	0.880
BMI, mean ± SD	29.59 ± 3.03	30.26 ± 3.36	29.79 ± 2.45	29.61 ± 2.57	28.57 ± 3.30	< 0.001
WC (cm) mean ± SD	98.62 ± 10.52	101.07 ± 10.43	99.96 ± 8.27	99.37 ± 8.65	93.71 ± 12.50	< 0.001
Total energy (kcal/day)	2099.78 ± 322.32	2106.5 ± 321.47	2126.7 ± 328.18	2059.9 ± 315.37	2099.05 ± 324.32	0.511
Carbohydrate (gr/day)	275.51 ± 66.39	269.14 ± 69.82	286.33 ± 65.51	275.21 ± 60.68	272.42 ± 67.41	0.220
Protein (gr/day)	69.32 ± 16.16	70.15 ± 17.19	69.16 ± 15.06	67.68 ± 15.18	69.94 ± 16.90	0.682
Fat (gr/day)	70.74 ± 15.29	72.23 ± 14.92	69.46 ± 16.01	70.51 ± 14.36	70.47 ± 15.81	0.555
Fiber (gr/day)	23.51 ± 14.65	19.80 ± 12.32	21.32 ± 16.09	21.82 ± 7.76	31.56 ± 17.17	< 0.001
**Dietary components of MIND score**						
Green leafy vegetables (serving/week)	5.05 ± 2.03	2.61 ± 0.61	4.44 ± 0.50	6.26 ± 0.46	7.48 ± 1.17	< 0.001
Other vegetables (serving/week)	7.08 ± 3.59	4.61 ± 1.08	7.40 ± 1.50	8.33 ± 3.85	8.61 ± 5.00	< 0.001
Nuts (serving/week)	4.61 ± 1.85	2.35 ± 0.55	4.00 ± 0.45	5.64 ± 0.41	6.99 ± 0.45	< 0.001
Olive oil (tablespoons)	1.31 ± 0.53	0.67 ± 0.16	1.14 ± 0.13	1.61 ± 0.11	1.99 ± 0.13	< 0.001
Whole grain (serving/day)	2.88 ± 1.16	1.46 ± 0.34	2.50 ± 0.28	3.53 ± 0.25	4.36 ± 0.28	< 0.001

Values are presented as mean ± SD or n (%). MIND, Mediterranean-DASH Intervention for Neurodegenerative Delay diet score; BMI, Body Mass Index; WC, Waist Circumference; MET_PHYS, Metabolic Equivalent Task for Physical Activity.

### Hepatic, cardiometabolic, and inflammatory biomarkers by MIND score quartiles

As shown in Table 2, participants with higher adherence to the MIND diet demonstrated significantly improved cardiometabolic profiles, including lower fasting glucose and insulin levels. Fasting glucose, fasting insulin, and HOMA-IR decreased significantly across quartiles (all *p* < 0.001), indicating improved insulin sensitivity with greater MIND adherence. Lipid profiles also improved with higher MIND scores. Triglycerides (TG) and total cholesterol (TC) decreased significantly (*p* < 0.001), while HDL-C increased (*p* < 0.001). Low-density lipoprotein cholesterol (LDL-C) showed significant trend (*p* < 0.001). Liver enzymes—ALT, AST, GGT, and ALP—were all significantly lower in participants with higher MIND scores (all *p* < 0.001), suggesting reduced hepatic injury and inflammation. Systemic inflammation markers, including CRP, LPS, and TMAO, were inversely associated with MIND diet adherence (all *p* < 0.001). The F/B ratio, a marker of gut dysbiosis, was significantly lower in those with higher MIND scores (*p* < 0.001), indicating a more balanced microbial profile. Although Shannon index of α-diversity did not differ significantly (*p* = 0.863), Simpson diversity index scores were significantly higher in the top two quartiles (*p* < 0.001), reflecting greater microbial evenness and richness among individuals with better dietary quality.

**Table 2:** Biochemical, liver-related, and gut microbiota profiles by MIND score quartiles (*N* = 450).

**Variables**	**Total (*N* = 450)**	**Q1 (*N* = 131)**	**Q2 (*N* = 112)**	**Q3 (*N* = 96)**	**Q4 (*N* = 111)**	***P*-value**
TG (mg/dl)	177.82 ± 54.93	238.17 ± 15.99	203.44 ± 13.20	149.79 ± 29.42	105.00 ± 7.04	< 0.001
LDL (mg/dl)	126.09 ± 24.85	154.44 ± 16.1	134.03 ± 6.71	112.04 ± 10.38	96.79 ± 4.05	< 0.001
TC (mg/dl)	209.61 ± 25.85	239.59 ± 10.15	220.22 ± 7.60	193.35 ± 9.23	177.58 ± 4.96	< 0.001
HDL (mg/dl)	40.25 ± 7.08	33.36 ± 2.69	37.74 ± 1.64	43.68 ± 3.11	47.96 ± 7.20	< 0.001
Diastolic blood pressure (mmHg)	73.73 ± 9.58	74.83 ± 9.43	73.45 ± 10.22	72.75 ± 8.58	73.57 ± 9.91	0.414
Systolic blood pressure (mmHg)	116.31 ± 15.70	117.21 ± 15.49	115.01 ± 15.52	117.22 ± 15.73	115.75 ± 16.22	0.649
Fasting Glucose (mg/dL)	103.91 ± 17.38	121.25 ± 11.88	112.24 ± 7.13	95.75 ± 6.22	82.11 ± 3.99	< 0.001
Fasting Insulin (μU/mL)	13.83 ± 6.20	18.57 ± 5.79	17.56 ± 2.38	11.08 ± 2.93	6.83 ± 2.67	< 0.001
HOMA-IR	4.01 ± 1.71	5.98 ± 0.76	4.64 ± 0.48	3.04 ± 0.75	1.86 ± 0.19	< 0.001
ALT (U/L)	61.81 ± 19.94	79.78 ± 5.03	75.65 ± 5.91	53.39 ± 11.74	33.91 ± 3.55	< 0.001
AST (U/L)	46.59 ± 14.90	57.45 ± 12.88	56.36 ± 5.44	42.57 ± 7.26	27.41 ± 3.55	< 0.001
GGT (U/L)	79.12 ± 19.09	97.74 ± 7.15	90.42 ± 6.95	70.24 ± 9.98	53.41 ± 3.76	< 0.001
ALP (U/L)	75.28 ± 13.83	90.98 ± 11.24	77.52 ± 4.53	69.81 ± 2.38	59.22 ± 4.57	< 0.001
CRP (mg/L)	4.78 ± 1.71	6.84 ± 0.75	5.30 ± 0.60	3.76 ± 0.57	2.68 ± 0.25	< 0.001
TMAO (μM)	21.90 ± 13.20	40.11 ± 6.21	20.05 ± 6.31	13.34 ± 1.36	9.66 ± 3.33	< 0.001
LPS	1.00 ± 0.39	1.44 ± 0.27	1.09 ± 0.13	0.76 ± 0.11	0.60 ± 0.23	< 0.001
F.B ratio	1.68 ± 0.83	2.68 ± 0.81	1.58 ± 0.38	1.06 ± 0.12	1.11 ± 0.24	< 0.001
Shannon index	2.98 ± 0.49	2.99 ± 0.49	2.94 ± 0.49	2.98 ± 0.51	2.99 ± 0.49	0.863
Simpson score index	0.26 ± 0.08	0.27 ± 0.10	0.34 ± 0.09	0.21 ± 0.01	0.23 ± 0.02	< 0.001

Values are presented as mean ± SD. TG, Triglycerides; LDL, Low-Density Lipoprotein; TC, Total Cholesterol; HDL, High-Density Lipoprotein; HOMA-IR, Homeostatic Model Assessment of Insulin Resistance; ALT/AST/GGT/ALP, Liver enzymes (Alanine Aminotransferase, Aspartate Aminotransferase, Gamma-Glutamyl Transferase, Alkaline Phosphatase); CRP, C-Reactive Protein; TMAO, Trimethylamine N-Oxide; LPS, Lipopolysaccharides; F/B ratio, Firmicutes-to-Bacteroidetes ratio.

### Hepatic steatosis and fibrosis by MIND score quartiles

As shown in Table 3, higher MIND diet adherence was significantly associated with lower hepatic fat accumulation and fibrosis severity. HSI and CAP scores decreased across quartiles (HSI: *p* < 0.001; CAP: *p* < 0.001). Similarly, the FIB-4 index declined significantly with increasing MIND scores (*p* < 0.001), indicating less advanced fibrosis. Ultrasound-based hepatic steatosis grade also improved with greater MIND adherence (*p* < 0.001), with the highest proportion of Grade 3 steatosis observed in the lowest quartile (Q1: 71%) and the lowest in the highest quartile (Q4: 13.5%).

**Table 3:** Hepatic biomarkers by MIND score quartiles (*N* = 450).

**Variables**	**Total (*N* = 450)**	**Q1 (*N* = 131)**	**Q2 (*N* = 112)**	**Q3 (*N* = 96)**	**Q4 (*N* = 111)**	***P*-value**
HSI	49.68 ± 8.38	59.59 ± 2.74	52.80 ± 2.70	44.87 ± 3.00	39.02 ± 1.07	< 0.001
FIB-4	2.07 ± 0.74	2.84 ± 0.50	2.42 ± 0.23	1.71 ± 0.25	1.15 ± 0.14	< 0.001
CAP score (dB/m)	258.38 ± 43.59	309.87 ± 14.27	274.60 ± 14.06	233.34 ± 15.60	202.90 ± 5.57	< 0.001
Grade hepatic steatosis, *n* (%)						< 0.001
Grade 1	139 (31)	14 (10.5)	8 (7)	37 (38.5)	80 (72)	
Grade 2	141 (31.5)	24 (18.3)	59 (59)	42 (44)	16 (14.5)	
Grade 3	170 (37.8)	93 (71)	45 (40)	17 (17.5)	15 (13.5)	

Values are presented as mean ± SD. HSI, Hepatic Steatosis Index; FIB-4, Fibrosis-4 Index; CAP, Controlled Attenuation Parameter.

### Mediation analysis: role of gut microbiota diversity

**Figure 2:**
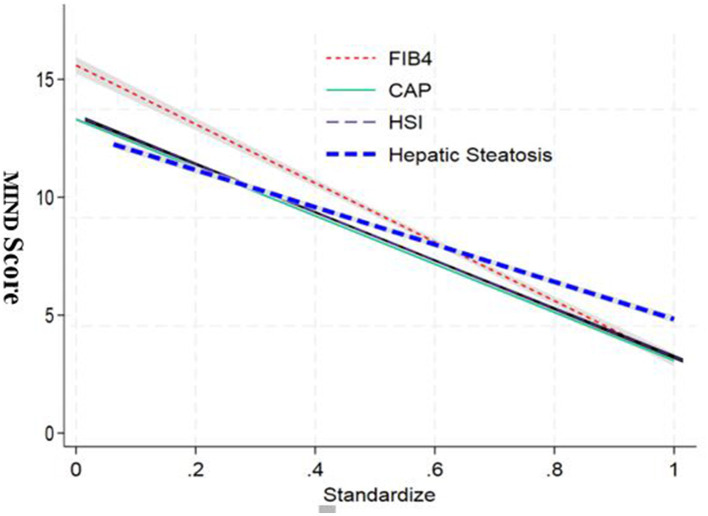
Mediation analysis of the association between MIND diet score and hepatic outcomes via simpson diversity index. Path diagram showing direct and indirect (mediated) effects of the MIND diet score on liver health indicators including hepatic fibrosis (FIB-4 index), hepatic steatosis (HSI), controlled attenuation parameter (CAP), and steatosis grade. Solid arrows represent direct paths; dashed arrows indicate mediated effects through Simpson diversity. All models were adjusted for age, sex, and BMI. Asterisks denote statistically significant pathways (*p* < 0.05).

Mediation analysis revealed that the Simpson diversity index partially mediated the relationship between MIND diet adherence and key hepatic outcomes, as presented in Table 4. The direct effect of MIND score on fibrosis, as estimated by FIB-4 index, was −0.18 (95% CI: −0.32 to −0.04), while the indirect effect through Simpson diversity was 0.06 (95% CI: 0.01 to 0.11). This resulted in a total effect of −0.12, indicating that while the majority of the influence of the MIND diet on fibrosis appears to be direct, a small but statistically significant portion is exerted through improvements in gut microbial diversity. Similarly, for the HSI, the direct effect of MIND score was −2.22 (95% CI: −2.25 to −2.19), and the indirect pathway via Simpson diversity was −0.02 (95% CI: −0.03 to 0), yielding a total effect of −2.24. In the case of Controlled Attenuation Parameter (CAP) score, the direct effect was −11.54 (95% CI: −11.72 to −11.37), with an indirect effect of −0.096 (95% CI: −0.17 to −0.02), resulting in a total effect of −11.64 (Figure 2). Lastly, for hepatic steatosis grade, the direct effect was −0.22 (95% CI: −0.23 to −0.22), and the indirect effect through Simpson diversity was 0.02 (95% CI: 0.01 to 0.03), leading to a total effect of −0.22. These results collectively suggest that although the predominant impact of higher MIND diet adherence on liver health is direct, there exists a measurable contribution of gut microbial diversity to this association, particularly in modulating markers of fibrosis and steatosis.

**Table 4:** Mediation analysis: role of Simpson diversity index in the relationship between MIND score and hepatic outcomes.

**Pathway**	**Effect type**	**β (95% CI)**
MIND score → FIB-4	Direct effect	−0.18 (−0.32, −0.04)
MIND score → Simpson score → FIB-4	Indirect effect	0.06 (0.01_0.11)
Total effect		–**0.12 (–0.26_0.02)**
MIND score → HSI	Direct effect	−2.22 (−2.25_−2.19)
MIND score → Simpson score → HSI	Indirect effect	−0.02 (−0.03_0)
Total effect		–**2.24 (–2.27_–2.21)**
MIND score → CAP score	Direct effect	−11.54 (−11.72_−11.37)
MIND score → Simpson score → CAP score	Indirect effect	−0.096 (−0.17_−0.02)
Total effect		**–11.64 (–11.8_–11.48)**
MIND score → Hepatic grade	Direct effect	−0.24 (−0.25_−0.23)
MIND score → Simpson score → Hepatic grade	Indirect effect	0.02 (0.01_0.03)
Total effect		**–0.22 (–0.23_–0.21)**

The table presents results from mediation analyses assessing the role of the Simpson Diversity Index in mediating the relationship between the MIND score and hepatic outcomes (FIB-4 index, HSI, CAP score, and Hepatic grade). Values are estimates with 95% confidence intervals in parentheses.

Direct effect: effect of MIND score on hepatic outcome not mediated through Simpson Diversity Index.

Indirect effect: effect of MIND score on hepatic outcome through Simpson Diversity Index.

Total effect: sum of direct and indirect effects.

Adjusted for age, sex, BMI, and physical activity; calorie and fiber intake.

FIB-4, Fibrosis-4 Index; his, Hepatic Steatosis Index; CAP, Controlled Attenuation Parameter. Bold values indicate statistically significant results at p < 0.05.

In additional mediation analyses, Simpson diversity was found to partially mediate the relationship between MIND score and CRP (Indirect effect: −0.28; 95% CI: −0.51 to −0.08) and LPS (Indirect effect: −0.04; 95% CI: −0.07 to −0.01), suggesting gut microbial balance contributes to anti-inflammatory effects.

Beyond overall diversity indices, participants in the highest MIND diet adherence tertile (Q3) exhibited significantly higher relative abundances of Lactobacillaceae, Ruminococcaceae, Bacteroidaceae, Faecalibacterium, and Bifidobacterium compared to those in the lowest tertile (Q1) (Supplementary Table S2). These taxa are commonly associated with improved gut microbial health, further supporting the beneficial impact of a high-quality dietary pattern on gut microbiota composition.

### Linear regression analysis: association between MIND score and hepatic, inflammatory, and cardiometabolic outcomes

The linear regression analysis further supported these findings by demonstrating consistent and significant inverse associations between MIND diet score and various hepatic, inflammatory, and cardiometabolic biomarkers, as detailed in Table 5. After adjusting for age, sex, physical activity (MET-minutes/week), and BMI, higher MIND scores were significantly linked to improved insulin sensitivity, as reflected by lower fasting insulin (β = −0.20; *p* < 0.001) and HOMA-IR (β = −2.11; *p* < 0.001). Liver enzymes were all significantly reduced with greater adherence to the MIND diet, with β values ranging from −0.17 to −0.26 across models. Additionally, systemic inflammation was inversely associated with MIND score, with CRP showing a strong negative relationship (β = −2.12; *p* < 0.001), as did LPS, a marker of metabolic endotoxemia (β = −8.52; *p* < 0.001). Regarding gut microbiota composition, both the F/B ratio, a marker of dysbiosis, and the Simpson diversity index showed significant improvement with higher MIND scores, with β values of −3.60 (*p* = 0.002) and −17.82 (*p* < 0.001), respectively. These associations remained largely unchanged after further adjustment for total calorie and fiber intake in Model 2, supporting the robustness of the observed effects. Given the number of biomarker comparisons conducted (*n* = 20), we applied Bonferroni correction to control for Type I error inflation. A corrected significance threshold of *p* < 0.0025 was used, and most associations retained statistical significance under this more conservative criterion, further reinforcing the strength and credibility of the observed relationships.

**Table 5:** Linear regression analysis of MIND score with hepatic, inflammatory, and cardiometabolic biomarkers.

**Dependent variables**	**Crude β(95%CI)**	**Model 1** **β(95%CI)**	**Model 2 β(95%CI)**	**Clinically significant**	**The Bonferroni adjustment**
Fasting Glucose (mg/dL)	−0.49(−0.52_ −0.46)	−0.50 (−0.53_ −0.46)	−0.49 (−0.52_ −0.45)	No	< 0.001
Fasting Insulin (μU/mL)	−0.20 (−0.21_ −0.20)	−0.20 (−0.21_ −0.20)	−0.20 (−0.21_ −0.19)	Yes	< 0.001
HOMA-IR	−2.11 (−2.15_ −2.07)	−2.11 (−2.15_ −2.07)	−2.10 (−2.14_ −2.06)	Yes	< 0.001
ALT (U/L)	−0.18 (−0.18_ −0.17)	−0.18 (−0.18_ −0.17)	−0.17 (−0.17_ −0.16)	No	< 0.001
AST (U/L)	−0.21 (−0.23_ −0.20)	−0.21 (−0.23_ −0.20)	−0.21 (−0.22_ −0.20)	No	< 0.001
GGT (U/L)	−0.19 (−0.19_ −0.18)	−0.19 (−0.19_ −0.18)	−0.18 (−0.19_ −0.17)	Yes	< 0.001
ALP (U/L)	−0.25 (−0.26_ −0.24)	−0.25 (−0.26_ −0.24)	−0.26 (−0.27_ −0.25)	Yes	< 0.001
CRP (mg/L)	−2.12 (−2.16_ −2.09)	−2.12 (−2.16_ −2.09)	−2.11 (−2.15_ −2.07)	Yes	< 0.001
TMAO (μM)	−0.25 (−0.26_ −0.24)	−0.25 (−0.26_ −0.24)	−0.24 (−0.25_ −0.23)	Yes	< 0.001
Total Cholesterol (mg/dl)	−0.14 (−0.14_ −0.14)	−0.14 (−0.14_ −0.14)	−0.14 (−0.14_ −0.13)	No	< 0.001
TG (mg/dl)	−0.07 (−0.07_ −0.06)	−0.07 (−0.07_ −0.06)	−0.06 (−0.06_ −0.06)	No	< 0.001
LDL (mg/dl)	−0.14 (−0.15_ −0.14)	−0.14 (−0.15_ −0.14)	−0.14 (−0.15_ −0.13)	No	< 0.001
TC (mg/dl)	−0.14 (−0.14_ −0.14)	−0.14 (−0.14_ −0.14)	−0.14 (−0.14_ −0.13)	No	< 0.001
HDL (mg/dl)	0.43 (0.41_ 0.46)	0.43 (0.41_ 0.46)	0.41 (0.39_ 0.44)	No	< 0.001
Diastolic blood pressure (mmHg)	−0.02 (−0.05_ 0.02)	−0.02 (−0.05_ 0.02)	−0.02 (−0.05_ 0.01)	No	< 0.001
Systolic blood pressure (mmHg)	−0.002 (−0.02_ 0.02)	−0.002 (−0.02_ 0.02)	−0.003 (−0.02_ 0.01)	No	0.41
HSI	−0.43 (−0.44_ −0.43)	−0.43 (−0.44_ −0.43)	−0.43 (−0.44_ −0.42)	Yes	0.93
LPS	−8.52 (−8.92_ −8.12)	−8.51 (−8.88_ −8.14)	−8.51 (−8.88_ −8.14)	Yes	< 0.001
TMAO	−0.25 (−0.26_ −0.24)	−0.25 (−0.26_ −0.24)	−0.24 (−0.25_ −0.23)	Yes	< 0.001
F/B ratio	−3.60 (−3.84_ −3.36)	−3.55 (−3.80_ −3.29)	−3.47 (−3.73_ −3.22)	Yes	0.002
Shannon index	0.12 (−0.58_ 0.81)	0.11 (−2.59_ 2.81)	−0.06 (−2.60_ 2.58)	Yes	0.01
Simpson index	−17.82 (−21.44_ −14.20)	−15.72 (−19.24_ −12.19)	−15.19 (−18.64_ −11.74)	Yes	< 0.001
FIB-4	−4.58 (−4.79, −4.36)	−4.53 (−4.74, −4.33)	−4.41 (−4.61, −4.21)	Yes	0.960
CAP	−0.08 (−0.08, −0.09)	−0.08 (−0.08, −0.08)	−0.08 (−0.08, −0.08)	No	0.620

Linear regression results are presented as beta coefficients (β) with 95% confidence intervals (CI).

Crude model: Unadjusted.

Model 1: Adjusted for age, sex, MET (metabolic equivalent task), and BMI.

Model 2: Model 1 further adjusted for total calorie and fiber intake.

Clinically significant: Defined as |β| ≥ 0.2 in magnitude for continuous biomarkers; for categorical or highly skewed biomarkers, clinical relevance was determined based on biological plausibility and prior literature.

Bonferroni adjustment: Indicates whether the association remained statistically significant after Bonferroni correction for multiple comparisons.

ALT, Alanine aminotransferase; AST, Aspartate aminotransferase; GGT, Gamma-glutamyl transferase; ALP, Alkaline phosphatase; CRP, C-reactive protein; TMAO, Trimethylamine N-oxide; F/B ratio, Firmicutes/Bacteroidetes ratio; HIS, Hepatic Steatosis Index; LPS, Lipopolysaccharide; FIB-4, Fibrosis-4 Index; CAP, Controlled Attenuation Parameter.

**Figure 3:**
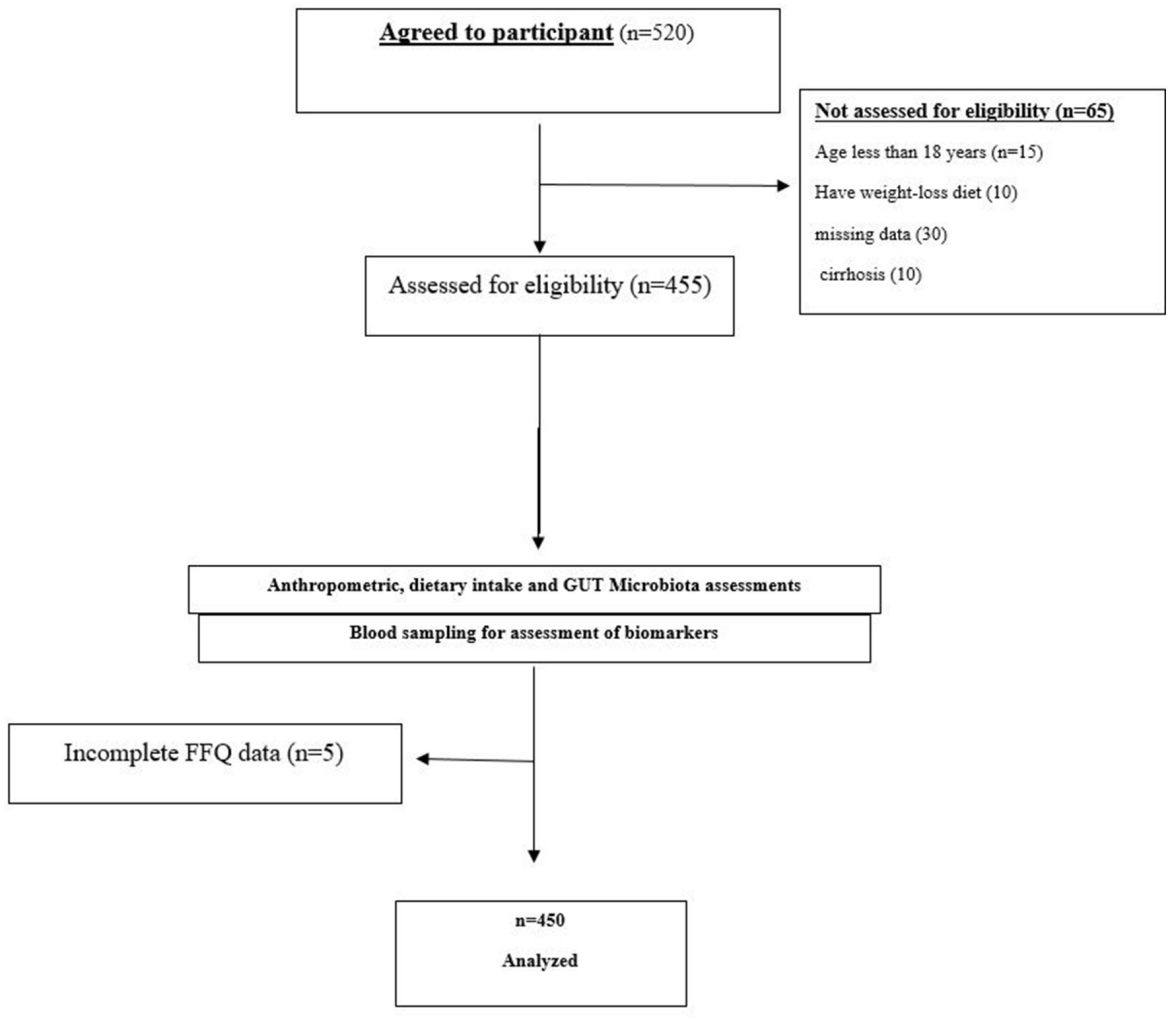
Standardized regression coefficients between MIND diet score and key clinical outcomes. Bar chart displaying β coefficients and 95% confidence intervals from multivariate linear regression models assessing the relationship between MIND score and hepatic (HSI, FIB-4, CAP), inflammatory (CRP, LPS), metabolic (HOMA-IR, insulin), and gut microbiota (Simpson index, F/B ratio) markers.

The regression analysis revealed significant associations between the standardized independent variables and the MIND score. As depicted in the graph, the FIB4 index showed a strong negative correlation with the MIND score. The adjusted standardization ensured that these relationships were not influenced by scale differences or outliers, providing a clear picture of the relative contributions of each variable (Figure 3).

Clinically meaningful associations, defined a priori as β coefficients with absolute values ≥ 0.2, were identified for several key outcomes including fasting insulin, HOMA-IR, GGT, ALP, CRP, TMAO, LPS, F/B ratio, and Simpson diversity index. These findings underscore the functional and clinical relevance of the MIND diet beyond statistical significance, particularly in relation to systemic inflammation, liver injury, and gut microbial balance (Table 5).

## Discussion

Our findings provide novel evidence that higher adherence to the MIND score is significantly associated with reduced severity of MASLD, including lower hepatic steatosis and fibrosis scores, improved insulin sensitivity, reduced systemic inflammation, and favorable gut microbial profiles. Importantly, this study represents the first to comprehensively evaluate the mediating role of gut microbiota diversity—particularly α-diversity as measured by the Simpson index—in linking dietary quality to hepatic outcomes among adults with MASLD. These observations provide novel insights into how dietary patterns influence multi-systemic health through interconnected physiological pathways, particularly those involving the gut-liver axis and systemic inflammation.

Participants in higher MIND quartiles had lower BMI and waist circumference, despite similar energy intake and physical activity levels. This suggests the MIND diet may improve body composition independent of caloric restriction. Greater adherence was also associated with higher intake of leafy greens, vegetables, whole grains, nuts, and olive oil—foods rich in fiber, antioxidants, and anti-inflammatory nutrients. These patterns reflect a high-quality, plant-focused diet consistent with known metabolic benefits (22–24).

Our study reveals a strong inverse relationship between adherence to the MIND diet and various indicators of liver injury and fat accumulation, such as liver enzymes (ALT, AST, GGT, ALP), HSI, CAP, and FIB-4. We found a dose-response relationship where higher MIND adherence correlates with improved markers of hepatic steatosis (HSI, CAP, ultrasound grade) and fibrosis (FIB-4 index). Notably, the lowest quartile showed a significantly higher prevalence of Grade 3 steatosis. This indicates that the MIND diet may not only help prevent but also reduce the progression of early-stage NAFLD, positioning it as a valuable nutritional strategy for both primary and secondary prevention. These associations remained robust even after adjusting for potential confounders such as age, sex, physical activity, and BMI, suggesting that the relationship between dietary quality and liver health is not merely a reflection of body weight differences. The biological plausibility of these findings is well-supported by previous literature indicating that dietary patterns rich in polyphenols, monounsaturated fatty acids, omega-3s, and low-glycemic index carbohydrates can reduce hepatic fat deposition and attenuate fibrotic processes (25, 26). Several components of the MIND diet—including olive oil, berries, whole grains, and nuts—have been shown to modulate hepatic lipogenesis, oxidative stress, and Kupffer cell activation, which are central to the pathogenesis of metabolic-associated steatotic liver disease (MASLD) (27, 28). Our results indicate that while the majority of the effect of the MIND diet on hepatic outcomes is direct—likely due to its rich content of polyphenols, fiber, and unsaturated fats—there exists a statistically significant, albeit smaller, indirect effect through increased gut microbial evenness (as captured by the Simpson index). This suggests that the MIND diet's influence on the liver extends beyond direct metabolic and anti-inflammatory effects and includes modulation of the gut ecosystem, reinforcing the importance of the gut-liver axis in dietary interventions for MASLD (29). The protective effects of the MIND diet on liver health appear to be multifactorial. At the core of the MIND pattern are foods rich in polyphenols (e.g., berries, green leafy vegetables), fiber (whole grains, legumes), and unsaturated fats (nuts, olive oil)—nutrients known to enhance insulin sensitivity, reduce oxidative stress, and suppress pro-inflammatory signaling (6, 12, 22, 26, 27). These components collectively may reduce *de novo* lipogenesis, promote fatty acid oxidation, and attenuate endoplasmic reticulum stress and mitochondrial dysfunction—all key drivers of hepatic steatosis and progression to steatohepatitis.

We also observed improvements in cardiometabolic markers including fasting glucose, insulin, HOMA-IR, triglycerides, and HDL-C. These effects suggest the MIND diet may exert systemic benefits that influence liver health indirectly through improved metabolic regulation (30).

Markers of systemic and gut-derived inflammation, including CRP, LPS, and TMAO, were significantly lower among those with greater MIND adherence. Lower LPS suggests improved gut barrier integrity, while reduced TMAO may reflect lower intake of red meat and higher plant-based food consumption, which alters microbial metabolism in favor of cardiometabolic health (31–33).

Beyond traditional metabolic and inflammatory markers, we explored the gut microbiota as a novel mediator in the diet–liver axis. Our data reveal that higher MIND diet scores were associated with a lower F/B ratio and greater Simpson diversity index values—both indicative of a healthier, more balanced microbial ecosystem. Although no significant differences were observed in Shannon α-diversity, the Simpson index—which emphasizes species evenness—revealed a more nuanced picture of microbial health. These findings suggest that the MIND diet may reshape gut microbial communities in ways that promote intestinal barrier integrity and reduce endotoxemia. Paragraph to Add: The gut-liver axis plays a pivotal role in MASLD pathogenesis, with diet-induced changes in microbial composition influencing hepatic health through multiple pathways. The MIND diet's high content of fiber and polyphenols likely promotes the growth of beneficial taxa such as Faecalibacterium and Bifidobacterium, which produce SCFAs like butyrate (34). SCFAs enhance gut barrier integrity, reducing bacterial translocation and LPS-mediated inflammation, which are key drivers of hepatic steatosis and fibrosis (35). Additionally, microbial metabolism of bile acids, modulated by dietary patterns, regulates hepatic lipid metabolism and inflammation via farnesoid X receptor signaling (34). These mechanisms underscore the therapeutic potential of the MIND diet in modulating the gut-liver axis to mitigate MASLD progression.

Given the growing recognition of the gut-liver axis in the pathogenesis of liver diseases, our findings provide important evidence linking dietary quality with microbial-mediated liver health (36, 37). Mechanisms such as bacterial translocation, short-chain fatty acid production, and bile acid metabolism are increasingly recognized as mediators of hepatic inflammation and fibrogenesis, and our data support the role of the MIND diet in modulating these pathways (34, 38).

The mediation analysis offered deeper insight into the interplay between diet, microbiota, and liver health. It demonstrated that Simpson diversity partially mediated the association between MIND diet adherence and key hepatic outcomes. While the magnitude of the indirect effects was relatively small compared to the direct effects of the diet, their statistical significance supports the hypothesis that microbial diversity functions as a functional intermediary in the MIND–liver relationship.

For instance, the indirect effects of Simpson diversity contributed to the observed relationships between MIND adherence and hepatic fibrosis (FIB-4 index), steatosis (HSI and CAP scores), and ultrasound-based steatosis grading. These findings align with emerging experimental and clinical evidence highlighting the gut–liver axis as a modifiable pathway influenced by dietary patterns, with direct implications for the prevention and management of MASLD (14, 39).

Effect sizes for key clinical outcomes (e.g., insulin, CRP, ALP, GGT, LPS, TMAO) exceeded the predefined threshold (β ≥ 0.2), suggesting these associations are not only statistically, but clinically meaningful. Consistent findings across multiple models support a dose–response relationship between MIND adherence and hepatic health, further validating these results.

This study has several notable strengths. First, it employs a comprehensive and multidimensional approach to assess hepatic outcomes, integrating clinical, biochemical, imaging-based, and microbial indicators. Second, it incorporates mediation and regression analyses to unpack potential mechanisms of action, adding interpretative depth to the observed associations. Third, it applies rigorous statistical control for confounding and multiple testing, reducing the likelihood of spurious findings. However, certain limitations must be acknowledged. The cross-sectional design limits causal inference, and the potential for residual confounding cannot be fully excluded. The lack of a healthy control group limits our ability to generalize findings beyond MASLD patients. Future case-control or longitudinal studies incorporating healthy individuals are warranted to validate these associations. Dietary data were self-reported, introducing the possibility of recall or reporting bias, although the use of a validated diet quality score mitigates this concern to some extent. Additionally, although liver stiffness measurement (LSM) via transient elastography provides a more direct assessment of fibrosis, only FIB-4 was available in our clinical dataset. CAP and LSM measurements were either missing or inconsistently recorded, limiting their use. This is acknowledged as a methodological limitation

## Conclusion

In summary, our findings suggest that higher adherence to the MIND diet is associated with significant improvements in hepatic steatosis, fibrosis, cardiometabolic function, systemic inflammation, and gut microbiota diversity. These benefits appear to be driven largely by direct dietary effects, with gut microbial diversity acting as a partial mediator. These results provide novel insight into the role of high-quality dietary patterns in liver health and support the integration of the MIND diet into dietary recommendations for MASLD prevention and management. Future longitudinal and intervention studies are warranted to confirm these associations and to explore the causal mechanisms underlying the observed effects.

## Data Availability

The raw data supporting the conclusions of this article will be made available by the authors, without undue reservation.
