# Supplementary material for: MIND diet score and its association with metabolic dysfunction-associated steatotic liver disease and gut microbiota profiles: a cross-sectional study
Source: Front Nutr. 2025 Aug 25;12:1637572. doi: 10.3389/fnut.2025.1637572 (PMC12414731; doi:10.3389/fnut.2025.1637572)
Supplement: Supplementary file 1 [file Table_1.docx]

**Dietary Assessment and MIND Scoring**

Dietary intake was assessed using a validated semi-quantitative food frequency questionnaire (FFQ) designed to capture habitual intake over the past year. The FFQ included standard portion sizes and frequency options and was administered by trained dietitians to minimize recall bias. The MIND diet score was computed based on 15 dietary components, adapted from the original scoring method developed by Morris et al., and modified for cultural dietary patterns and data availability in this study.

The 15 components included 10 brain-healthy food groups (green leafy vegetables, other vegetables, nuts, berries, legumes, whole grains, fish, poultry, olive oil, and wine) and 5 brain-unhealthy food groups (red meats, butter and stick margarine, cheese, pastries and sweets, and fast/fried food).

To account for differences in energy intake, all dietary components were adjusted using the residual method. Participants were then categorized into tertiles of intake for each component. For brain-healthy food groups, participants in the highest tertile received a score of 1, those in the middle tertile received 0.5, and those in the lowest tertile received 0. For brain-unhealthy food groups, the scoring was reversed: individuals in the highest tertile received a score of 0, those in the middle tertile received 0.5, and those in the lowest tertile received 1, indicating lower intake of harmful foods.

Each component was scored individually, and the total MIND diet score was obtained by summing the scores across all components. As a result, total scores ranged from 0 (no adherence) to 15 (maximum adherence). For descriptive analyses, participants were categorized into low and high adherence groups using a predefined cutoff: scores <8.5 indicated low adherence, and scores ≥9 indicated high adherence.

**Supplementary File 1: Food Frequency Questionnaire (FFQ) and MIND Diet Scoring Protocol**

**Section A: Food Frequency Questionnaire (FFQ) Overview**

Participants were asked to report their habitual intake of specific food items over the past year using a semi-quantitative FFQ. The FFQ included 110 food items, grouped into the following major categories relevant to MIND diet scoring:

1. **Green leafy vegetables** (e.g., spinach, kale, lettuce)
2. **Other vegetables** (e.g., carrots, broccoli, tomatoes)
3. **Nuts** (e.g., almonds, walnuts, pistachios)
4. **Berries** (e.g., blueberries, strawberries, mulberries)
5. **Legumes** (e.g., lentils, chickpeas, kidney beans)
6. **Whole grains** (e.g., whole wheat bread, oats, brown rice)
7. **Fish** (e.g., sardines, salmon, mackerel)
8. **Poultry** (e.g., chicken, turkey)
9. **Olive oil** (used for cooking or dressing)
10. **Wine** (e.g., red or white, as part of meals)
11. **Red meats** (e.g., beef, lamb, pork)
12. **Butter and stick margarine**
13. **Cheese**
14. **Pastries and sweets** (e.g., cakes, cookies, candies)
15. **Fast/fried food** (e.g., French fries, fried chicken, hamburgers)

Participants reported the frequency of consumption for each item on the following scale:

- Never or less than once per month
- 1–3 times per month
- 1–2 times per week
- 3–4 times per week
- 5–6 times per week
- Once per day
- 2 or more times per day

Portion sizes were standardized using visual aids.

**Section B: MIND Diet Scoring Protocol**

The MIND diet scoring was based on 15 components, including wine. Each participant's intake of the components was adjusted for energy using the residual method. Tertiles of intake were generated from the population distribution.

**Scoring System:**

- For each **brain-healthy** component:
  - Highest tertile: 1 point
  - Middle tertile: 0.5 point
  - Lowest tertile: 0 point
- For each **brain-unhealthy** component:
  - Lowest tertile: 1 point
  - Middle tertile: 0.5 point
  - Highest tertile: 0 point

**Total Score Range:** 0 to 15

Each component score was summed to generate the overall MIND diet score for each participant.

**Section C: Sample FFQ**

| **Food Item Category** | **Example Items** | **Frequency Response** |
| --- | --- | --- |
| Green leafy vegetables | Spinach, kale, lettuce | Once per day |
| Other vegetables | Carrots, broccoli | 2 or more times per day |
| Nuts | Almonds, walnuts | 5–6 times per week |
| Berries | Blueberries, strawberries | 3–4 times per week |
| Legumes | Lentils, kidney beans | Once per day |
| Whole grains | Brown rice, whole wheat bread | 2 or more times per day |
| Fish | Salmon, sardines | 3–4 times per week |
| Poultry | Chicken, turkey | Once per day |
| Olive oil | Used in cooking | Daily use |
| Wine | Red/white wine with meals | 1–2 times per week |
| Red meats | Beef, lamb | 1–2 times per week |
| Butter/stick margarine |  | 1–3 times per month |
| Cheese |  | 1–3 times per month |
| Pastries and sweets | Cakes, cookies | Less than once/month |
| Fast/fried food | Fried chicken, fries | Never |

**Section D: Sample Scoring Calculation for a High-Adherence Participant**

| **Component** | **Tertile Category** | **Score Assigned** |
| --- | --- | --- |
| Green leafy vegetables | Highest | 1 |
| Other vegetables | Highest | 1 |
| Nuts | Highest | 1 |
| Berries | Highest | 1 |
| Legumes | Highest | 1 |
| Whole grains | Highest | 1 |
| Fish | Middle | 0.5 |
| Poultry | Highest | 1 |
| Olive oil | Highest | 1 |
| Wine | Middle | 0.5 |
| Red meats | Lowest | 1 |
| Butter/margarine | Lowest | 1 |
| Cheese | Lowest | 1 |
| Pastries and sweets | Lowest | 1 |
| Fast/fried food | Lowest | 1 |

**Total MIND Score:** 13.0 points
