# Supplementary material for: MIND diet score and its association with metabolic dysfunction-associated steatotic liver disease and gut microbiota profiles: a cross-sectional study
Source: Front Nutr. 2025 Aug 25;12:1637572. doi: 10.3389/fnut.2025.1637572 (PMC12414731; doi:10.3389/fnut.2025.1637572)
Supplement: Supplementary file 2 [file Table_2.docx]

| **Supplementary Table S2. Relative Abundance of Selected Gut Microbial Taxa Across MIND Diet Score Tertiles** | | | | |
| --- | --- | --- | --- | --- |
| **Taxa** | **Q1 (Lowest)Mean % ± SD** | **Q2 (Middle)Mean % ± SD** | **Q3 (Highest)Mean % ± SD** | **P-value** |
| **Family Level** |  |  |  |  |
| *Ruminococcaceae* | 13.68 ± 0.55 | 15.70 ± 0.69 | 18.01 ± 0.59 | <0.001 |
| *Lactobacillaceae* | 4.85 ± 0.55 | 6.87 ± 0.69 | 9.18 ± 0.59 | <0.001 |
| *Bacteroidaceae* | 21.47 ± 0.55 | 23.49 ± 0.69 | 25.80 ± 0.59 | <0.001 |
| **Genus Level** |  |  |  |  |
| *Faecalibacterium* | 11.45 ± 1.30 | 16.21 ± 1.64 | 21.67 ± 1.39 | <0.001 |
| *Bifidobacterium* | 3.64 ± 0.41 | 5.15 ± 0.52 | 6.89 ± 0.44 | <0.001 |
| Values are presented as mean relative abundance (%) ± standard deviation.  P-values are based on one-way ANOVA or Kruskal-Wallis test depending on data normality.  Post-hoc pairwise comparisons were performed where significant to identify group differences. | | | | |
